# Association of plain water intake with self-reported depression and suicidality among Korean adolescents

**DOI:** 10.4178/epih.e2024019

**Published:** 2024-01-09

**Authors:** Jung Woo Lee, Yookyung Kim

**Affiliations:** 1 BK21 Four Research & Education Center for Sustainable Living System, Korea University, Seoul, Korea; 2 Department of Home Economics Education, Korea University College of Education, Seoul, Korea

**Keywords:** Adolescent, Depression, Mental health, Drinking water, Suicide

## Abstract

**OBJECTIVES:**

Adolescent depression and suicidality are serious health problems worldwide. Lower plain water intake has been proposed as a risk factor for depression in adults. This study investigated the association of daily plain water intake with self-reported depression and suicidality among Korean adolescents.

**METHODS:**

We used nationwide data from 112,250 students aged 12-18 years who participated in the Korean Youth Risk Behavior Web-based Surveys in 2019 and 2020. Daily plain water intake was categorized as <1 glass, 1-2 glasses, and ≥3 glasses. The adjusted odds ratios (aORs) with 95% confidence intervals (CIs) for self-reported depression and suicidality were calculated using multiple regression analyses.

**RESULTS:**

The weighted prevalence rates of self-reported depression, suicidal ideation, suicide planning, and suicide attempts were 26.7%, 12.0%, 3.8%, and 2.5%, respectively. Of the participants, 3.9%, 18.5%, and 77.7% were categorized into the <1 glass/day, 1-2 glass/day, and ≥3 glass/day groups, respectively. Compared to the reference group (≥3 glass/day), the lowest level of water intake (<1 glass/day) was associated with higher odds of self-reported depression (aOR, 1.30; 95% CI, 1.20 to 1.39), suicidal ideation (aOR, 1.39; 95% CI, 1.27 to 1.55), suicide planning (aOR, 1.46; 95% CI, 1.25 to 1.69), and suicide attempts (aOR, 1.38; 95% CI, 1.15 to 1.67). Moderately lower water intake (1-2 glass/day) showed slightly increased odds of self-reported depression (aOR, 1.05; 95% CI, 1.01 to 1.10) and suicidal ideation (aOR, 1.08; 95% CI, 1.03 to 1.14).

**CONCLUSIONS:**

Lower plain water intake was significantly associated with a higher risk of self-reported depression and suicidality among Korean adolescents. Since this cross-sectional study is unable to establish a causal relationship, it underscores the need for additional longitudinal research.

## INTRODUCTION

The global prevalence of adolescent mental disorders is approximately 14% [1]. Depressive and anxiety disorders are the leading causes of morbidity among adolescents of both genders [2]. Adolescent depression is a well-known risk factor for substance abuse, risky behavioral problems, and suicidality [3-5]. A prospective naturalistic study showed that major depressive disorder was associated with a 5.5-fold higher risk of suicide attempts between adolescence and young adulthood [6]. Suicide is the major cause of adolescent deaths in many countries, including Korea [7]. Therefore, the early detection and management of adolescent depression and factors influencing depression may play a crucial role in preventing suicide among young people. Recent evidence suggests a relationship between unhealthy dietary behaviors and depression. An “unhealthy diet” includes fast foods or takeaways, foods with high fat and sugar levels, confectionery, sweetened beverages, fried foods, processed meat, and baked products [8,9].

A growing body of evidence has shown that water balance can affect mood and cognition [10-12]. Controlled human trials have found that dehydration can impair mood [13,14]. A French intervention study demonstrated that increased daily water intake could lead to significant mood improvements in habitual low-water drinkers [15]. However, limited information is available regarding the association between plain water intake and mental disorders. A recent study from Iran found that men and women who drank less than 2 glasses of water per day had 73% and 54% higher risks of depression, respectively [16]. The study included 3,327 adults working at universities in 20 cities across an Iranian province, warranting the need for further validation of the results in various settings and populations, including adolescents. Therefore, using nationwide population data, the present study aimed to investigate the association of daily plain water intake with self-reported depression and suicidality among Korean adolescents.

## MATERIALS AND METHODS

### Design, data, and study population

We performed a cross-sectional population study using data from 12-year-old to 18-year-old students who participated in the Korean Youth Risk Behavior Web-based Survey (KYRBS) conducted in 2019 and 2020. The KYRBS is a nationwide, school-based, annual survey conducted by the Korea Disease Control and Prevention Agency (KDCA) [17]. The KYRBS, established in 2005, was designed to assess Korean adolescents’ health behaviors and to plan health promotion projects for them. The KYRBS used a multi-stage cluster sampling design to obtain a nationally representative sample of Korean students. Data were collected using an anonymous web-based self-reporting questionnaire. The survey had 103-105 questions that assessed 16 domains of health-risk behavior, including dietary behavior, obesity and weight control, physical activity, sleep, self-reported depression and suicidality, smoking, alcohol intake, and sexual intercourse. Plain water intake has been measured since 2019 as a dietary behavior.

The participation rates were 95.3% (57,303/60,100) and 94.9% (54,948/57,925) in 2019 and 2020, respectively. Data from the 2019-2020 KYRBS were combined according to the instructions provided by the KDCA. Of the 112,251 survey participants during 2019-2020, 1 participant was excluded because of a lack of data. Finally, data from 112,250 participants were included in this study, corresponding to 2.1% of the entire Korean student population aged between 12 years and 18 years.

### Measurements

#### Plain water intake

Plain water intake was a key independent variable. Plain water was defined as still water, non-sweetened sparkling water, or barley tea. Plain water intake levels were measured using a questionnaire asking, “How often have you been drinking plain water in the last seven days?” The responses were categorized as ≥ 5 glass/day, 4 glass/day, 3 glass/day, 1-2 glass/day, and < 1 glass/day. One glass of water was designated as 200 mL. According to the 2020 Dietary Reference Intakes for Koreans [18], which recommends drinking 610-920 mL plain water per day for 12-year-old to 18-year-old adolescents, we recategorized water intake levels into ≥ 3 glass/day, 1-2 glass/day, and < 1 glass/day.

#### Perceived depression and suicidality

The dependent variables used in this study were self-reported depression, suicidal ideation, suicide planning, and suicide attempts. Each variable was assessed using a single question with a dichotomous response (yes or no). The following questions were asked: “In the past 12 months, have you felt sadness or despair enough to stop your daily activities for at least two weeks?” (self-reported depression); “Have you seriously considered suicide in the last 12 months?” (suicidal ideation); “Have you ever made any specific plans for suicide in the last 12 months?” (suicide planning); “Have you ever attempted suicide in the last 12 months?” (suicide attempts).

#### Covariates

The confounding covariates included gender, age, body mass index (BMI), type of school, economic status, academic achievement, physical activity, smoking, alcohol consumption, carbonated beverage intake, and sweetened beverage intake. The BMI was calculated as weight (kg)/height (m^2^). School type was classified into middle school (reference group) and high school. Economic status and academic achievement were categorized as high (reference group), middle, or low. Physical activity was categorized as ≥ 4 day/wk (vigorous physical activity for at least 60 min/day: reference group), 1-3 day/wk, and none. Smoking status was categorized as none (never smoked in their lifetime or no smoking in the last month: reference group) or current smoking. Alcohol consumption was categorized as none (lifetime abstainer or no history of alcohol consumption in the previous month: reference group) or current alcohol consumption. Carbonated beverage intake was categorized into 4 groups according to the drinking frequency in the last 7 days: none (reference group), < 2 times/wk, 3-6 times/wk, and daily. Sweetened beverage intake was categorized into 4 groups according to drinking frequency in the last 7 days: none (reference group), < 2 times/wk, 3-6 times/wk, and daily.

### Statistical analysis

Data were reported as percentages with standard errors for categorical variables and as means with standard errors for continuous variables. Covariate variables across the categories of plain water intake levels were tested using the chi-square test or oneway analysis of variance. Multiple logistic regression was used to estimate the adjusted odds ratio (aOR) and 95% confidence interval (CI) for both self-reported depression and suicidality. We included the confounding covariates of gender, age, BMI, type of school, economic status, academic achievement, physical activity, smoking status, alcohol consumption status, carbonated beverage intake, and sweetened beverage intake in the adjusted models. All data were analyzed by applying survey sampling weights according to KDCA’s statistical guidelines. All statistical analyses were performed using SPSS version 28.0 (IBM Corp., Armonk, NY, USA). A p-value < 0.05 indicated a statistically significant result.

### Ethics statement

The Institutional Review Board of the KDCA approved the KYRBS. Informed consent was obtained from all the participants. We only used publicly available data.

## RESULTS

### Prevalence of self-reported depression and suicidality

**Figure 1.**
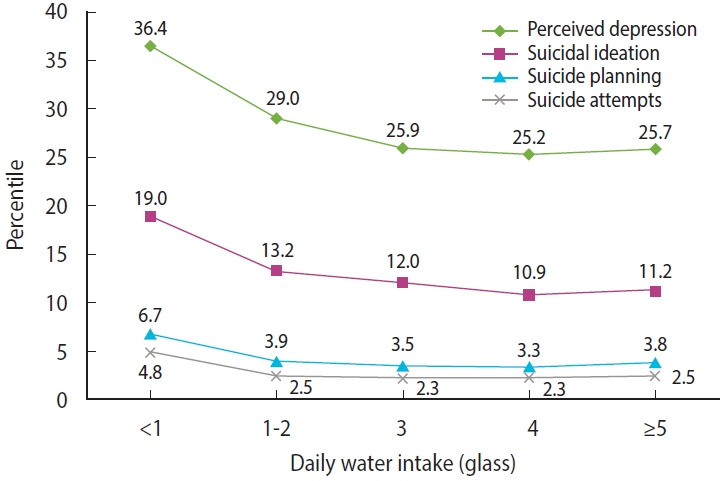
The weighted prevalences of self-reported depression, suicidal ideation, suicide planning, and suicide attempts according to daily water intake.

The weighted prevalence rates of self-reported depression, suicidal ideation, suicide planning, and suicide attempts were 26.7%, 12.0%, 3.8%, and 2.5%, respectively. Figure 1 and Supplementary Material 1 show the prevalence according to the daily water intake. The highest prevalence of depression and suicidality was observed in the group with the lowest water intake (< 1 glass/day), followed by the group with 1-2 glass/day intake. The prevalence of depression and suicidality then plateaued in the groups with intake of 3 glass/day, 4 glass/day, and ≥ 5 glass/day. When the participants were re-categorized into 3 groups based on their water intake (< 1, 1-2, and ≥ 3 glass/day), the proportion of participants in each group was 3.9%, 18.5%, and 77.7%, respectively.

### General characteristics according to daily plain water intake

Table 1 presents the general characteristics of the participants according to their daily plain water intake. Of the 112,250 participants, 51.9% were men, and 48.1% were women. The mean age was 15.1 years. The percentage of middle and high school students was 48.7% and 51.3%, respectively. The proportion of women students was higher in the <1 glass/day group (75.9%) and 1-2 glass/day group (64.4%) than in the ≥ 3 glass/day group (42.8%). Mean age; the proportions of low economic status, low academic achievement, and no physical activity; and the daily intake of carbonated beverages and sweetened beverages were highest in the < 1 glass/day group and lowest in the ≥ 3 glass/day group (p< 0.001). The mean BMI was the lowest in the < 1 glass/day group. Current smoking and alcohol consumption were most common in the < 1 glass/day group, whereas they were the least common in the 1-2 glass/day group.

### Association of daily plain water intake with self-reported depression and suicidality

Table 2 shows the ORs and 95% CIs for the associations between self-reported depression and suicidality across daily plain water intake categories. In the unadjusted model, the lowest water intake (< 1 glass/day) was associated with substantial increases in the odds of self-reported depression (aOR, 1.66; 95% CI, 1.55 to 1.77), suicidal ideation (aOR, 1.82; 95% CI, 1.68 to 1.98), suicide planning (aOR, 1.92; 95% CI, 1.66 to 2.21), and suicide attempts (aOR, 2.06; 95% CI, 1.75 to 2.44). After adjusting for covariates, the associations remained significant for self-reported depression (aOR, 1.30; 95% CI, 1.20 to 1.39), suicidal ideation (aOR, 1.39; 95% CI, 1.27 to 1.55), suicide planning (aOR, 1.46; 95% CI, 1.25 to 1.69), and suicide attempts (aOR, 1.38; 95% CI, 1.15 to 1.67). Sex-specific analyses showed similar trends in both men and women participants (Supplementary Materials 2 and 3). When stratified by physical activity, the highest physical activity group (≥ 4 day/wk) had the strongest associations for self-reported depression (aOR, 1.44; 95% CI, 1.11 to 1.86), suicidal ideation (aOR, 2.03; 95% CI, 1.51 to 2.74), suicide planning (aOR, 1.68; 95% CI, 1.09 to 2.59), and suicide attempts (aOR, 2.00; 95% CI, 1.22 to 3.28) (Supplementary Material 4).

The moderately lower water intake group (1-2 glass/day) had slightly increased odds of self-reported depression (aOR, 1.05; 95% CI, 1.01 to 1.10) and suicidal ideation (aOR, 1.08; 95% CI, 1.03 to 1.14) in both the unadjusted and adjusted models. Suicide planning was significantly associated with daily 1-2 glass/day plain water intake in the unadjusted model (OR, 1.10; 95% CI, 1.01 to 1.20). However, this was not significant in the adjusted model (aOR, 1.08; 95% CI, 0.98 to 1.18). Suicide attempts did not show a significant association in the unadjusted (OR, 1.07; 95% CI, 0.97 to 1.19) or adjusted models (aOR, 1.03; 95% CI, 0.92 to 1.16).

### Association of daily water intake and beverage intake with self-reported depression and suicidality

**Figure 2.**
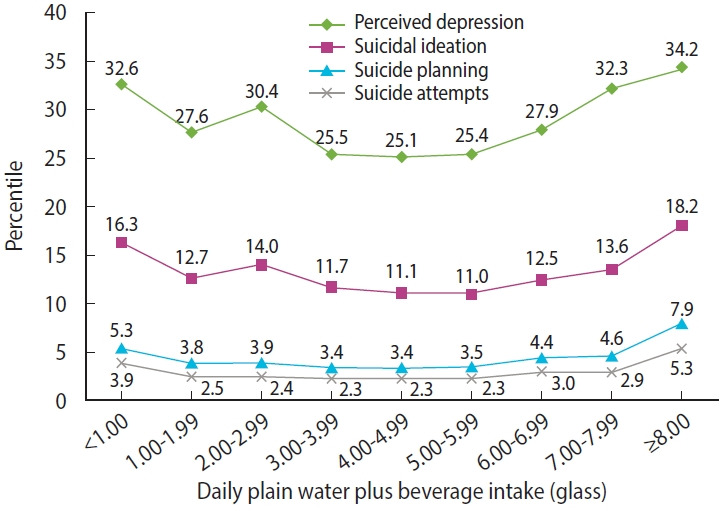
The weighted prevalences of self-reported depression, suicidal ideation, suicide planning, and suicide attempts according to daily water and beverage intake.

**Figure 3.**
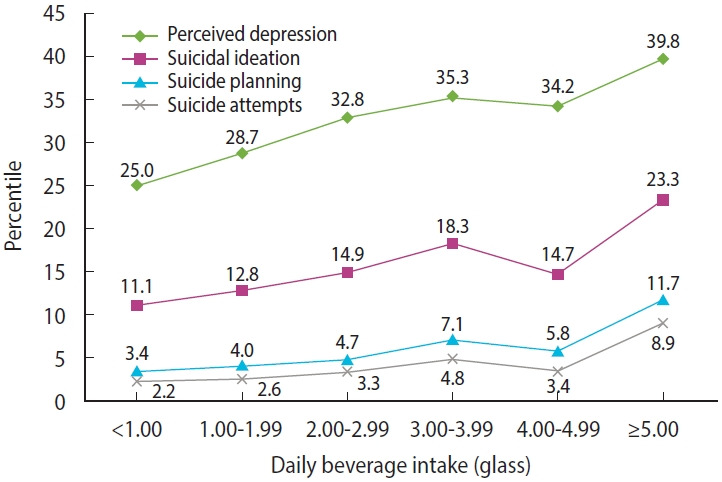
The weighted prevalences of self-reported depression, suicidal ideation, suicide planning, and suicide attempts according to daily beverage intake.

Figure 2 and Supplementary Material 5 show the prevalence rates of the outcome variables based on the combination of daily water intake and consumption of beverages (including carbonated- and sweetened beverages). The graphs reveal a U-shaped pattern in the relationship between the prevalence of the outcome variables and combined daily water and beverage intake. Stratification according to physical activity showed similar trends across all the groups (Supplementary Material 6). When examining the graph solely based on beverage consumption, an upward trend aligns with an increase in self-reported depression and suicidality (Figure 3, Supplementary Material 7).

### Association of covariates with self-reported depression and suicidality

Supplementary Material 8 shows the prevalence of self-reported depression, suicidal ideation, suicide planning, and suicide attempts according to the covariates. Table 3 summarizes the aORs and 95% CIs for the associations of the other covariates with self-reported depression and suicidality. Women gender, middle school, low economic status, low academic achievement, current smoking, and daily intake of sweetened beverages were significantly associated with increased risks of self-reported depression, suicidal ideation, suicide planning, and suicide attempts (p< 0.05).

## DISCUSSION

This cross-sectional study investigated the association of plain water intake with self-reported depression and suicidality among Korean adolescents. Using data from a nationwide population-based study, we showed that lower plain water intake was significantly associated with a higher risk of self-reported depression and suicidality, in an inverse dose-dependent pattern. To the best of our knowledge, this is the first study to examine the impact of plain water intake on mental health problems among adolescents.

It is still uncertain why a lack of water intake was found to be associated with a higher risk of self-reported depression and suicidality. Dehydration caused by low water intake is a possible explanation. Imbalances in brain neurotransmitters or hormones such as serotonin, dopamine, and norepinephrine have long been considered to contribute to the development of depression [19]. Studies have shown that water deprivation can alter the levels of these molecules [20-22]. Well-controlled human studies have demonstrated that restricted water intake significantly and negatively impairs mood states and sleep parameters [15,23]. Notably, even mild dehydration affects mood states and cognitive function [13,14], and mood changes caused by dehydration may not be rapidly reversed by *ad libitum* fluid intake [23]. The impact of dehydration particularly concerns those with poor water balance regulation, such as children or older adults [24]. Additionally, dehydration can disrupt the balance of electrolytes in the body, which may lead to neuropsychiatric symptoms ranging from confusion to depression to suicidal ideation [25]. This study, however, examined the association of plain water intake with self-reported depression and suicidality. An important factor to consider when explaining this relationship through dehydration is that it did not take into account the amount of hydration provided by beverages and food. A U-shaped pattern was identified in the association between self-reported depression, suicidality, and the combined daily intake of plain water and beverages. We speculate that the U-shaped pattern may have resulted from the combined effects of plain water intake and beverage consumption. If there were a causal relationship between plain water intake and self-reported depression and suicidality, it would hold significant implications from a public health perspective. This could involve the potential use of screening tests to identify depression and suicidality indicators, as well as considering therapeutic interventions. The need for continued, rigorous research in this field is emphasized.

Despite the potential impact of plain water intake on mood, the direct causal relationship between plain water intake and depression or suicidality remains largely unproven in the context of established mental disorders. Therefore, there is a high likelihood that the relationship between plain water intake and depression or suicidality may not be a straightforward causal relationship. Instead of being a direct cause of depression or suicidality, decreased water intake might serve as a symptom or indicator of various interconnected factors. For instance, it could be a manifestation of depression itself, as individuals experiencing depression might neglect self-care practices, including proper hydration. Moreover, inadequate water intake may also signal an overall suboptimal dietary pattern, potentially lacking in essential nutrients crucial for mental well-being. A French group reported that drinking water intake is associated with a higher diet quality [26]. In our study, low water intake was associated with various unhealthy behaviors, including no active physical activity, higher carbonated beverage intake, and higher sweetened beverage intake. Low water intake is often associated with high consumption of sugar-sweetened beverages, as individuals may opt for such drinks instead of water as their primary source of hydration [27]. Cumulative evidence has shown a positive association between high sugar-sweetened beverage consumption and depression in adolescents [28-30]. Commercially available sugar-sweetened beverages typically contain high levels of sugar and fructose. High consumption of sugar-sweetened beverages may increase the risk of obesity, type 2 diabetes, and metabolic syndrome [31], which have been shown to increase the risk of depression. High fructose consumption may also contribute to depression by altering the levels of inflammatory cytokines or certain hormones, such as leptin and ghrelin, which regulate appetite and mood [32]. It is crucial to emphasize that this cross-sectional study can only provide evidence of an association between water intake and self-reported feelings of depression and suicidality. Consequently, additional longitudinal research is essential to substantiate these preliminary findings and reach a deeper comprehension of the underlying mechanisms at play.

We found no significant differences in the prevalence of self-reported depression and suicidality among adolescents who consumed 3 glass/day, 4 glass/day, or ≥ 5 glass/day. This finding aligns with the minimum water intake recommendation for Korean adolescents, which is about 3 glass/day [18]. An Iranian study categorized daily water intake as < 2 glass/day, 2-5 glass/day, and ≥ 5 glass/day. Compared to the 5 glass/day group, both the < 2 glass/day group and 2-5 glass/day group exhibited a higher likelihood of having depression in the fully adjusted model (< 2 glass/day group: aOR, 1.79; 2-5 glass/day group: aOR, 1.37) [16]. Since the participants in that study were all adults, it is natural that the categorization criteria differed from those of adolescents. Even in adolescents, the minimum water intake recommendation may differ according to individual characteristics, such as body weight or gender. This warrants a further detailed analysis.

It is worth considering whether we should actively encourage plain water intake in adolescents with low drinking water intake. In a recent study, American investigators have shown that adolescents who did not consume plain water consumed nearly twice the calories from sugar-sweetened beverages [27]. The authors suggested that adolescents drink water daily to help avoid excess calorie and sugar intake. It is uncertain whether plain water has therapeutic effects; however, it is safe and can potentially reduce the intake of sugary drinks, making it a recommended option. Given the accumulating evidence linking high consumption of sugar-sweetened beverages to depression in adolescents, it would be helpful to educate and guide them toward consuming more plain water as part of their daily intake rather than relying on excessive drinks containing sugar or fructose.

The current study has several limitations. First, because water intake was measured through self-report questionnaires and not through a detailed analysis of dietary intake, the reported amount of water intake may not be accurate. Second, the KYRBS survey only included adolescents who were school students. The results among non-student adolescents may differ from those of adolescent students. The high school enrollment rates in Korea were 91.4% in 2019 and 90.8% in 2020 [33]. Third, as described earlier, this cross-sectional study could not establish a causal relationship between water intake and adolescents’ mental health. Finally, the diagnosis of depression and suicidality in this study was based on self-reported data and not through a formal diagnosis by mental health professionals.

In conclusion, daily plain water intake was found to be significantly associated with the risk of self-reported depression and suicidality in Korean adolescents. However, given the limitations of this cross-sectional study in determining causation, further longitudinal research is imperative.

## Figures and Tables

**Table 1.** General characteristics of participants according to their daily plain water intake

Characteristics	Total (n=112,250)	<1 glass/day (n=4,301)	1-2 glass/day (n=20,776)	3-5 glass/day (n=87,173)	p-value
Weighted %	100 (0.0)	3.9 (0.1)	18.5 (0.2)	77.7 (0.2)	
Gender					
Men	51.9 (0.9)	24.1 (1.0)	35.6 (1.0)	57.2 (0.9)	
Women	48.1 (0.9)	75.9 (1.0)	64.4 (1.0)	42.8 (0.9)	<0.001
Age, mean (yr)	15.1 (0.0)	15.4 (0.0)	15.3 (0.0)	15.1 (0.0)	<0.001
Body mass index, mean (kg/m^2^)	21.4 (0.0)	20.1 (0.1)	20.6 (0.0)	21.7 (0.0)	<0.001
Type of school					
Middle	48.7 (0.6)	41.0 (1.4)	43.6 (0.7)	50.3 (0.6)	
High	51.3 (0.6)	59.0 (1.1)	56.4 (0.7)	49.7 (0.6)	<0.001
Economic status					
High	39.8 (0.3)	33.4 (0.8)	34.8 (0.4)	41.3 (0.3)	
Middle	47.6 (0.2)	50.1 (0.9)	51.4 (0.4)	46.6 (0.3)	
Low	12.6 (0.2)	16.5 (0.6)	13.8 (0.3)	12.1 (0.2)	<0.001
Academic achievement					
High	37.5 (0.2)	30.8 (0.8)	34.7 (0.4)	38.5 (0.3)	
Middle	30.4 (0.2)	27.5 (0.7)	30.8 (0.4)	30.1 (0.2)	
Low	32.4 (0.2)	41.7 (0.8)	34.5 (0.4)	31.4 (0.2)	<0.001
Physical activity (day/wk)					
≥4	20.6 (0.2)	7.6 (0.5)	10.1 (0.3)	23.8 (0.2)	
1-3	42.1 (0.2)	31.3 (0.8)	41.1 (0.4)	42.9 (0.2)	
None	37.3 (0.3)	61.2 (0.9)	48.8 (0.5)	33.4 (0.3)	<0.001
Smoking					
None	94.4 (0.1)	94.1 (0.4)	95.8 (0.2)	94.1 (0.2)	
Current	5.6 (0.1)	5.9 (0.4)	4.2 (0.2)	5.9 (0.2)	<0.001
Alcohol consumption					
None	87.2 (0.2)	86.5 (0.6)	88.2 (0.3)	86.9 (0.2)	
Current	12.9 (0.2)	13.5 (0.6)	11.8 (0.3)	13.1 (0.2)	<0.001
Carbonated beverage intake					
None	21.2 (0.2)	20.9 (0.7)	20.3 (0.4)	21.5 (0.2)	
≤2/wk	42.5 (0.2)	38.1 (0.8)	42.2 (0.4)	42.9 (0.2)	
3-6/wk	29.6 (0.2)	27.8 (0.8)	30.5 (0.4)	29.5 (0.2)	
Daily	6.6 (0.1)	13.2 (0.6)	7.1 (0.2)	6.2 (0.1)	<0.001
Sweetened beverage intake					
None	14.5 (0.1)	13.0 (0.6)	12.6 (0.3)	15.1 (0.2)	
≤2/wk	37.3 (0.2)	30.4 (0.8)	36.8 (0.4)	37.8 (0.2)	
3-6/wk	37.9 (0.2)	37.0 (0.8)	38.9 (0.4)	37.7 (0.2)	
Daily	10.2 (0.1)	19.6 (0.7)	11.7 (0.3)	9.4 (0.1)	<0.001
Perceived depression					
No	73.3 (0.2)	63.6 (0.8)	71.0 (0.4)	74.3 (0.2)	
Yes	26.7 (0.2)	36.4 (0.8)	29.0 (0.4)	25.7 (0.2)	<0.001
Suicidal ideation					
No	88.0 (0.1)	81.0 (0.6)	86.8 (0.3)	88.6 (0.1)	
Yes	12.0 (0.1)	19.0 (0.6)	13.2 (0.3)	11.4 (0.1)	<0.001
Suicide planning					
No	96.2 (0.1)	93.3 (0.4)	96.1 (0.2)	96.4 (0.1)	
Yes	3.8 (0.1)	6.7 (0.4)	3.9 (0.2)	3.6 (0.1)	<0.001
Suicide attempts					
No	97.5 (0.1)	95.2 (0.4)	97.5 (0.1)	97.6 (0.1)	
Yes	2.5 (0.1)	4.8 (0.4)	2.5 (0.1)	2.4 (0.1)	<0.001

Values are presented as weighted percentage (standard error) unless otherwise stated.

**Table 2.** Odds ratios and 95% confidence intervals for associations of perceived depression and suicidality across daily plain water intake categories^1^

Variables	<1 glass/day	1-2 glass/day	≥3 glass/day	p-value
Perceived depression				
Crude model	1.66 (1.55, 1.77)	1.18 (1.14, 1.23)	1.00 (reference)	<0.001
Model 1	1.35 (1.26, 1.45)	1.04 (1.00, 1.09)	1.00 (reference)	<0.001
Model 2	1.28 (1.19, 1.38)	1.03 (0.99, 1.07)	1.00 (reference)	<0.001
Model 3	1.30 (1.20, 1.39)	1.05 (1.01, 1.10)	1.00 (reference)	<0.001
Suicidal ideation				
Crude model	1.82 (1.68, 1.98)	1.19 (1.13, 1.25)	1.00 (reference)	<0.001
Model 1	1.51 (1.38, 1.65)	1.10 (1.04, 1.15)	1.00 (reference)	<0.001
Model 2	1.42 (1.30, 1.55)	1.07 (1.02, 1.13)	1.00 (reference)	<0.001
Model 3	1.39 (1.27, 1.55)	1.08 (1.03, 1.14)	1.00 (reference)	<0.001
Suicide planning				
Crude model	1.92 (1.66, 2.21)	1.10 (1.01, 1.20)	1.00 (reference)	<0.001
Model 1	1.60 (1.38, 1.86)	1.07 (0.98, 1.18)	1.00 (reference)	<0.001
Model 2	1.48 (1.28, 1.73)	1.05 (0.95, 1.15)	1.00 (reference)	<0.001
Model 3	1.46 (1.25, 1.69)	1.08 (0.98, 1.18)	1.00 (reference)	<0.001
Suicide attempts				
Crude model	2.06 (1.75, 2.44)	1.07 (0.97, 1.19)	1.00 (reference)	<0.001
Model 1	1.52 (1.27, 1.82)	1.01 (0.91, 1.13)	1.00 (reference)	<0.001
Model 2	1.37 (1.14, 1.64)	0.98 (0.87, 1.09)	1.00 (reference)	<0.001
Model 3	1.38 (1.15, 1.67)	1.03 (0.92, 1.16)	1.00 (reference)	<0.001

1 Model 1: Adjusted for gender, age, and body mass index; Model 2: Further adjusted for type of school, economic status, and academic achievement; Model 3: Further adjusted for smoking, alcohol consumption, physical activity, carbonated beverage intake, and sweetened beverage intake.

**Table 3.** Adjusted odds ratios and 95% confidence intervals for associations of covariates with perceived depression and suicidality^1^

Variables	Perceived depression	p-value	Suicidal ideation	p-value	Suicide planning	p-value	Suicidal attempts	p-value
Gender								
Men	1.00 (reference)		1.00 (reference)		1.00 (reference)		1.00 (reference)	
Women	2.10 (2.02, 2.18)	<0.001	2.20 (2.09, 2.32)	<0.001	1.94 (1.78, 2.12)	<0.001	2.83 (2.54, 3.16)	<0.001
Age	1.05 (1.03, 1.07)	<0.001	0.98 (0.96, 1.01)	0.160	0.96 (0.92, 0.99)	0.017	0.90 (0.86, 0.95)	<0.001
Body mass index	1.00 (1.00, 1.01)	0.224	1.03 (1.02, 1.04)	<0.001	1.03 (1.02, 1.04)	<0.001	1.03 (1.02, 1.04)	<0.001
Type of school								
Middle	1.00 (reference)		1.00 (reference)		1.00 (reference)		1.00 (reference)	
High	0.89 (0.83, 0.94)	<0.001	0.79 (0.72, 0.86)	<0.001	0.67 (0.60, 0.79)	<0.001	0.67 (0.56, 0.80)	<0.001
Economic status								
High	1.00 (reference)		1.00 (reference)		1.00 (reference)		1.00 (reference)	
Middle	0.95 (0.92, 0.99)	0.007	1.03 (0.98, 1.08)	0.310	0.95 (0.88, 1.04)	0.255	0.93 (0.84, 1.03)	0.183
Low	1.52 (1.45, 1.59)	<0.001	1.96 (1.84, 2.09)	<0.001	1.97 (1.78, 2.18)	<0.001	2.06 (1.82, 2.33)	<0.001
Academic achievement								
High	1.00 (reference)		1.00 (reference)		1.00 (reference)		1.00 (reference)	
Middle	1.07 (1.03, 1.11)	0.001	0.98 (0.93, 1.03)	0.467	1.03 (0.93, 1.13)	0.581	1.14 (1.02, 1.28)	0.028
Low	1.35 (1.30, 1.40)	<0.001	1.24 (1.18, 1.31)	<0.001	1.35 (1.24, 1.48)	<0.001	1.58 (1.40, 1.77)	<0.001
Physical activity (day/wk)								
≥4	1.00 (reference)		1.00 (reference)		1.00 (reference)		1.00 (reference)	
1-3	0.94 (0.90, 0.97)	0.001	0.99 (0.93, 1.05)	0.620	0.94 (0.85, 1.03)	0.185	0.83 (0.74, 0.93)	0.002
None	0.77 (0.73, 0.80)	<0.001	0.94 (0.88, 0.99)	0.030	0.85 (0.77, 0.94)	0.001	0.72 (0.64, 0.82)	<0.001
Smoking								
None	1.00 (reference)		1.00 (reference)		1.00 (reference)		1.00 (reference)	
Current	1.58 (1.47, 1.70)	<0.001	1.68 (1.53, 1.84)	<0.001	1.83 (1.60, 2.10)	<0.001	2.66 (2.28, 3.09)	<0.001
Alcohol consumption								
None	1.00 (reference)		1.00 (reference)		1.00 (reference)		1.00 (reference)	
Current	1.76 (1.67, 1.85)	<0.001	1.84 (1.73, 2.00)	<0.001	1.99 (1.79, 2.20)	<0.001	2.54 (2.24, 2.87)	<0.001
Carbonated beverage intake								
None	1.00 (reference)		1.00 (reference)		1.00 (reference)		1.00 (reference)	
≤2/wk	0.96 (0.93, 1.00)	0.071	0.90 (0.85, 0.96)	<0.001	0.79 (0.72, 0.87)	<0.001	0.81 (0.72, 0.92)	<0.001
3-6/wk	1.07 (1.02, 1.12)	0.006	1.00 (0.94, 1.07)	0.961	0.85 (0.76, 0.94)	0.002	0.97 (0.85, 1.11)	0.619
Daily	1.10 (1.02, 1.18)	0.011	1.05 (0.95, 1.16)	0.324	1.03 (0.88, 1.20)	0.752	1.06 (0.87, 1.29)	0.574
Sweetened beverage intake								
None	1.00 (reference)		1.00 (reference)		1.00 (reference)		1.00 (reference)	
≤2/wk	1.02 (0.97, 1.08)	0.386	0.97 (0.90, 1.03)	0.294	0.90 (0.80, 1.00)	0.053	0.94 (0.81, 1.08)	0.352
3-6/wk	1.18 (1.12, 1.24)	<0.001	1.11 (1.04, 1.19)	0.001	1.05 (0.94, 1.17)	0.414	0.96 (0.83, 1.11)	0.572
Daily	1.50 (1.40, 1.60)	<0.001	1.38 (1.27, 1.51)	<0.001	1.35 (1.18, 1.55)	<0.001	1.27 (1.06, 1.52)	0.010

1 Adjusted for all other variables shown in this table.

## Supplementary materials

Supplementary Material 1. Association of plain water intake with perceived depression and suicidality.

Supplementary Material 2. Odds ratios and 95% confidence intervals for associations of perceived depression and suicidality across daily plain water intake categories in male participants.

Supplementary Material 3. Odds ratios and 95% confidence intervals for associations of perceived depression and suicidality across daily plain water intake categories in female participants.

Supplementary Material 4. Odds ratios and 95% confidence intervals for perceived depression and suicidality by daily plain water intake categories, stratified by physical activity.

Supplementary Material 5. Association of plain water and beverage1 intake with perceived depression and suicidality.

Supplementary Material 6. The weighted prevalences of perceived depression, suicidal ideation, suicide planning, and suicide attempts according to the daily water and beverage intake, stratified by physical activity.

Supplementary Material 7. Association of beverage1 intake with perceived depression and suicidality.

Supplementary Material 8. Prevalences of perceived depression and suicidality according to the covariates.
