# Supplementary material for: Association of plain water intake with self-reported depression and suicidality among Korean adolescents
Source: Epidemiol Health. 2024 Jan 9;46:e2024019. doi: 10.4178/epih.e2024019 (PMC11099597; doi:10.4178/epih.e2024019)
Supplement: Supplementary Material 1. — Association of plain water intake with perceived depression and suicidality. [file epih-46-e2024019-Supplementary-1.docx]

**Supplementary Material 1.** Association of plain water intake with perceived depression and suicidality.^1^

| Variable | Total  N = 112,250 | < 1 glass/day  n = 4,301 | 1-2 glasses/day  n = 20,776 | 3 glasses/day  n = 25,149 | 4 glasses/day  n = 19,926 | 5 glasses/day  n = 42,098 |
| --- | --- | --- | --- | --- | --- | --- |
| Perceived depression |  |  |  |  |  |  |
| No | 73.3 (0.2) | 63.6 (0.8) | 71.0 (0.4) | 74.1 (0.3) | 74.8 (0.4) | 74.3 (0.3) |
| Yes | 26.7 (0.2) | 36.4 (0.8) | 29.0 (0.4) | 25.9 (0.3) | 25.2 (0.4) | 25.7 (0.3) |
| Suicidal ideation |  |  |  |  |  |  |
| No | 88.0 (0.1) | 81.0 (0.6) | 86.8 (0.3) | 88.0 (0.2) | 89.1 (0.2) | 88.8 (0.2) |
| Yes | 12.0 (0.1) | 19.0 (0.6) | 13.2 (0.3) | 12.0 (0.2) | 10.9 (0.2) | 11.2 (0.2) |
| Suicide planning |  |  |  |  |  |  |
| No | 96.2 (0.1) | 93.3 (0.4) | 96.1 (0.2) | 96.5 (0.1) | 96.7 (0.1) | 96.2 (0.1) |
| Yes | 3.8 (0.1) | 6.7 (0.4) | 3.9 (0.2) | 3.5 (0.1) | 3.3 (0.1) | 3.8 (0.1) |
| Suicide attempts |  |  |  |  |  |  |
| No | 97.5 (0.1) | 95.2 (0.4) | 97.5 (0.1) | 97.7 (0.1) | 97.7 (0.1) | 97.5 (0.1) |
| Yes | 2.5 (0.1) | 4.8 (0.4) | 2.5 (0.1) | 2.3 (0.1) | 2.3 (0.1) | 2.5 (0.1) |

^1^Data are presented as weighted percentage (standard error).
