# Supplementary material for: Association of plain water intake with self-reported depression and suicidality among Korean adolescents
Source: Epidemiol Health. 2024 Jan 9;46:e2024019. doi: 10.4178/epih.e2024019 (PMC11099597; doi:10.4178/epih.e2024019)
Supplement: Supplementary Material 2. — Odds ratios and 95% confidence intervals for associations of perceived depression and suicidality across daily plain water intake categories in male participants. [file epih-46-e2024019-Supplementary-2.docx]

**Supplementary Material 2.** Odds ratios and 95% confidence intervals for associations of perceived depression and suicidality across daily plain water intake categories in male participants.

|  | Adjusted odds ratio  (95% confidence interval) | |  |  |
| --- | --- | --- | --- | --- |
|  | < 1 glass/day | 1-2 glasses/day | ≥ 3 glasses/day | *p* |
| Perceived depression |  |  |  |  |
| Crude model | 1.33 (1.15-1.55) | 1.07 (1.00-1.14) | 1 | < 0.001 |
| Model 1^1^ | 1.24 (1.06-1.45) | 1.06 (0.99-1.13) | 1 | < 0.001 |
| Model 2^2^ | 1.18 (1.00-1.38) | 1.04 (0.98-1.11) | 1 | < 0.001 |
| Model 3^3^ | 1.18 (1.01-1.39) | 1.09 (1.02-1.16) | 1 | < 0.001 |
| Suicidal ideation |  |  |  |  |
| Crude model | 1.77 (1.45-2.17) | 1.11 (1.00-1.22) | 1 | < 0.001 |
| Model 1^1^ | 1.55 (1.23-1.94) | 1.14 (1.04-1.26) | 1 | < 0.001 |
| Model 2^2^ | 1.45 (1.16-1.81) | 1.12 (1.01-1.24) | 1 | < 0.001 |
| Model 3^3^ | 1.36 (1.08-1.70) | 1.13 (1.02-1.25) | 1 | < 0.001 |
| Suicide planning |  |  |  |  |
| Crude model | 2.41 (1.77-3.28) | 1.14 (0.97-1.33) | 1 | < 0.001 |
| Model 1^1^ | 1.85 (1.27-2.70) | 1.22 (1.03-1.45) | 1 | <0.001 |
| Model 2^2^ | 1.71 (1.18-2.49) | 1.20 (1.01-1.42) | 1 | <0.001 |
| Model 3^3^ | 1.57 (1.08-2.30) | 1.23 (1.04-1.46) | 1 | <0.001 |
| Suicide attempts |  |  |  |  |
| Crude model | 3.38 (2.45-4.66) | 1.01 (0.82-1.25) | 1 | < 0.001 |
| Model 1^1^ | 2.33 (1.56-3.47) | 1.12 (0.90-1.40) | 1 | < 0.001 |
| Model 2^2^ | 2.14 (1.42-3.20) | 1.10 (0.88-1.37) | 1 | < 0.001 |
| Model 3^3^ | 1.94 (1.29-2.93) | 1.17 (0.93-1.46) | 1 | < 0.001 |

^1^Model 1: adjusted for age and body mass index. ^2^Model 2: further adjusted for type of school, economic status, and academic achievement. ^3^Model 3: further adjusted for smoking, alcohol consumption, physical activity, carbonated beverage intake, and sweetened beverage intake.
