# Supplementary material for: Association of plain water intake with self-reported depression and suicidality among Korean adolescents
Source: Epidemiol Health. 2024 Jan 9;46:e2024019. doi: 10.4178/epih.e2024019 (PMC11099597; doi:10.4178/epih.e2024019)
Supplement: Supplementary Material 3. — Odds ratios and 95% confidence intervals for associations of perceived depression and suicidality across daily plain water intake categories in female participants. [file epih-46-e2024019-Supplementary-3.docx]

**Supplementary Material 3.** Odds ratios and 95% confidence intervals for associations of perceived depression and suicidality across daily plain water intake categories in female participants.

|  | Adjusted odds ratio  (95% confidence interval) | |  |  |
| --- | --- | --- | --- | --- |
|  | < 1 glass/day | 1-2 glasses/day | ≥ 3 glasses/day | *p* |
| Perceived depression |  |  |  |  |
| Crude model | 1.40 (1,29-1.51) | 1.04 (0.99-1.09) | 1 | < 0.001 |
| Model 1^1^ | 1.39 (1.28-1.51) | 1.04 (0.99-1.10) | 1 | < 0.001 |
| Model 2^2^ | 1.32 (1.21-1.43) | 1.03 (0.98-1.08) | 1 | < 0.001 |
| Model 3^3^ | 1.32 (1.21-1.43) | 1.04 (0.99-1.09) | 1 | < 0.001 |
| Suicidal ideation |  |  |  |  |
| Crude model | 1.44 (1.31-1.58) | 1.02 (0.96-1.08) | 1 | < 0.001 |
| Model 1^1^ | 1.53 (1.39-1.68) | 1.08 (1.02-1.15) | 1 | < 0.001 |
| Model 2^2^ | 1.43 (1.30-1.58) | 1.06 (1.00-1.12) | 1 | < 0.001 |
| Model 3^3^ | 1.42 (1.28-1.57) | 1.07 (1.01-1.14) | 1 | < 0.001 |
| Suicide planning |  |  |  |  |
| Crude model | 1.48 (1.26-1.74) | 0.94 (0.84-1.04) | 1 | < 0.001 |
| Model 1^1^ | 1.57 (1.33-1.85) | 1.02 (0.91-1.13) | 1 | <0.001 |
| Model 2^2^ | 1.44 (1.22-1.70) | 0.99 (0.88-1.10) | 1 | <0.001 |
| Model 3^3^ | 1.43 (1.21-1.70) | 1.02 (0.91-1.13) | 1 | <0.001 |
| Suicide attempts |  |  |  |  |
| Crude model | 1.38 (1.14-1.66) | 0.89 (0.79-1.01) | 1 | < 0.001 |
| Model 1^1^ | 1.41 (1.16-1.71) | 0.98 (0.86-1.11) | 1 | < 0.001 |
| Model 2^2^ | 1.25 (1.03-1.52) | 0.94 (0.83-1.07) | 1 | < 0.001 |
| Model 3^3^ | 1.28 (1.04-1.58) | 1.00 (0.88-1.14) | 1 | < 0.001 |

^1^Model 1: adjusted for age and body mass index. ^2^Model 2: further adjusted for type of school, economic status, and academic achievement. ^3^Model 3: further adjusted for smoking, alcohol consumption, physical activity, carbonated beverage intake, and sweetened beverage intake.
