# Supplementary material for: Association of plain water intake with self-reported depression and suicidality among Korean adolescents
Source: Epidemiol Health. 2024 Jan 9;46:e2024019. doi: 10.4178/epih.e2024019 (PMC11099597; doi:10.4178/epih.e2024019)
Supplement: Supplementary Material 4. — Odds ratios and 95% confidence intervals for perceived depression and suicidality by daily plain water intake categories, stratified by physical activity. [file epih-46-e2024019-Supplementary-4.docx]

**Supplementary Material 4.** Odds ratios and 95% confidence intervals for perceived depression and suicidality by daily plain water intake categories, stratified by physical activity.

|  | Adjusted odds ratio  (95% confidence interval) | |  |  |
| --- | --- | --- | --- | --- |
|  | < 1 glass/day | 1-2 glasses/day | ≥ 3 glasses/day | *P* value |
| **≥ 4 days/week** |  |  |  |  |
| Perceived depression |  |  |  |  |
| Crude model | 2.23 (1.76-2.83) | 1.23 (1.11-1.36) | 1 | < 0.001 |
| Adjusted model^1^ | 1.44 (1.11-1.86) | 1.07 (0.95-1.19) | 1 | 0.015 |
| Suicidal ideation |  |  |  |  |
| Crude model | 3.16 (2.42-4.14) | 1.25 (1.09-1.44) | 1 | < 0.001 |
| Adjusted model^1^ | 2.03 (1.51-2.74) | 1.10 (0.95-1.27) | 1 | < 0.001 |
| Suicide planning |  |  |  |  |
| Crude model | 3.14 (2.18-4.53) | 1.18 (0.94-1.49) | 1 | < 0.001 |
| Adjusted model^1^ | 1.68 (1.09-2.59) | 1.00 (0.78-1.28) | 1 | 0.065 |
| Suicide attempts |  |  |  |  |
| Crude model | 4.33 (2.93-6.40) | 1.11 (0.84-1.47) | 1 | < 0.001 |
| Adjusted model^1^ | 2.00 (1.22-3.28) | 0.89 (0.64-1.22) | 1 | 0.015 |
| **1-3 day(s)/week** |  |  |  |  |
| Perceived depression |  |  |  |  |
| Crude model | 1.83 (1.63-2.06) | 1.25 (1.18-1.32) | 1 | < 0.001 |
| Adjusted model^1^ | 1.44 (1.27-1.64) | 1.10 (1.04-1.17) | 1 | < 0.001 |
| Suicidal ideation |  |  |  |  |
| Crude model | 1.84 (1.58-2.13) | 1.15 (1.07-1.25) | 1 | < 0.001 |
| Adjusted model^1^ | 1.41 (1.20-1.66) | 1.06 (0.98-1.15) | 1 | < 0.001 |
| Suicide planning |  |  |  |  |
| Crude model | 1.81 (1.40-2.33) | 1.09 (0.95-1.26) | 1 | < 0.001 |
| Adjusted model^1^ | 1.42 (1.08-1.85) | 1.05 (0.90-1.21) | 1 | 0.041 |
| Suicide attempts |  |  |  |  |
| Crude model | 1.89 (1.39-2.56) | 1.08 (0.92-1.27) | 1 | < 0.001 |
| Adjusted model^1^ | 1.26 (0.90-1.78) | 0.97 (0.82-1.16) | 1 | 0.353 |
| **None** |  |  |  |  |
| Perceived depression |  |  |  |  |
| Crude model | 1.56 (1.42-1.71) | 1.15 (1.09-1.21) | 1 | < 0.001 |
| Adjusted model^1^ | 1.19 (1.08-1.32) | 1.00 (0.95-1.07) | 1 | 0.002 |
| Suicidal ideation |  |  |  |  |
| Crude model | 1.63 (1.46-1.83) | 1.17 (1.09-1.27) | 1 | < 0.001 |
| Adjusted model^1^ | 1.32 (1.17-1.49) | 1.10 (1.02-1.19) | 1 | < 0.001 |
| Suicide planning |  |  |  |  |
| Crude model | 1.90 (1.57-2.30) | 1.12 (0.99-1.27) | 1 | < 0.001 |
| Adjusted model^1^ | 1.48 (1.20-1.83) | 1.13 (0.99-1.30) | 1 | < 0.001 |
| Suicide attempts |  |  |  |  |
| Crude model | 2.00 (1.60-2.50) | 1.14 (0.97-1.34) | 1 | < 0.001 |
| Adjusted model^1^ | 1.41 (1.09-1.83) | 1.16 (0.97-1.37) | 1 | 0.017 |

^1^adjusted for sex, age, body mass index, type of school, economic status, academic achievement, smoking, alcohol consumption, physical activity, carbonated beverage intake, and sweetened beverage intake.
