# Supplementary material for: Association of plain water intake with self-reported depression and suicidality among Korean adolescents
Source: Epidemiol Health. 2024 Jan 9;46:e2024019. doi: 10.4178/epih.e2024019 (PMC11099597; doi:10.4178/epih.e2024019)
Supplement: Supplementary Material 5. — Association of plain water and beverage1 intake with perceived depression and suicidality. [file epih-46-e2024019-Supplementary-5.docx]

**Supplementary Material 5.** Association of plain water and beverage^1^ intake with perceived depression and suicidality.^2^

| Variable | Total  N = 112,250 | < 1 glass/day  n = 2,389 | 1-1.99 glasses/day  n = 9,233 | 2-2.99 glasses/day  n = 10,238 | 3-3.99 glasses/day  n = 18,524 | 4-4.99 glasses/day  n = 21,267 | 5-5.99 glasses/day  n = 34,558 | 6.-6.99 glasses/day  n = 12,052 | 7-7.99 glasses/day  n = 1,873 | ≥ 8 glasses/day  n = 2,116 |
| --- | --- | --- | --- | --- | --- | --- | --- | --- | --- | --- |
| Perceived depression | 26.7 (0.2) | 32.6 (1.0) | 27.6 (0.5) | 30.4 (0.5) | 25.5 (0.4) | 25.1 (0.4) | 25.4 (0.3) | 27.9 (0.5) | 32.3 (1.1) | 34.2 (1.1) |
| Suicidal ideation | 12.0 (0.1) | 16.3 (0.8) | 12.7 (0.4) | 14.0 (0.4) | 11.7 (0.3) | 11.1 (0.3) | 11.0 (0.2) | 12.5 (0.3) | 13.6 (0.9) | 18.2 (1.0) |
| Suicide planning | 3.8 (0.1) | 5.3 (0.5) | 3.8 (0.2) | 3.9 (0.2) | 3.4 (0.1) | 3.4 (0.1) | 3.5 0.1) | 4.4 (0.2) | 4.6 (0.5) | 7.9 (0.7) |
| Suicide attempts | 2.5 (0.1) | 3.9 (0.4) | 2.5 (0.2) | 2.4 (0.2) | 2.3 (0.1) | 2.3 (0.1) | 2.3 (0.1) | 3.0 (0.2) | 2.9 (0.4) | 5.3 (0.5) |

^1^Carbonated beverage plus sweetened beverage. ^2^Data are presented as weighted percentage (standard error).
