# Supplementary material for: Association of plain water intake with self-reported depression and suicidality among Korean adolescents
Source: Epidemiol Health. 2024 Jan 9;46:e2024019. doi: 10.4178/epih.e2024019 (PMC11099597; doi:10.4178/epih.e2024019)

**Supplementary Material 6.** The weighted prevalences of perceived depression, suicidal ideation, suicide planning, and suicide attempts according to the daily water and beverage intake, stratified by physical activity.


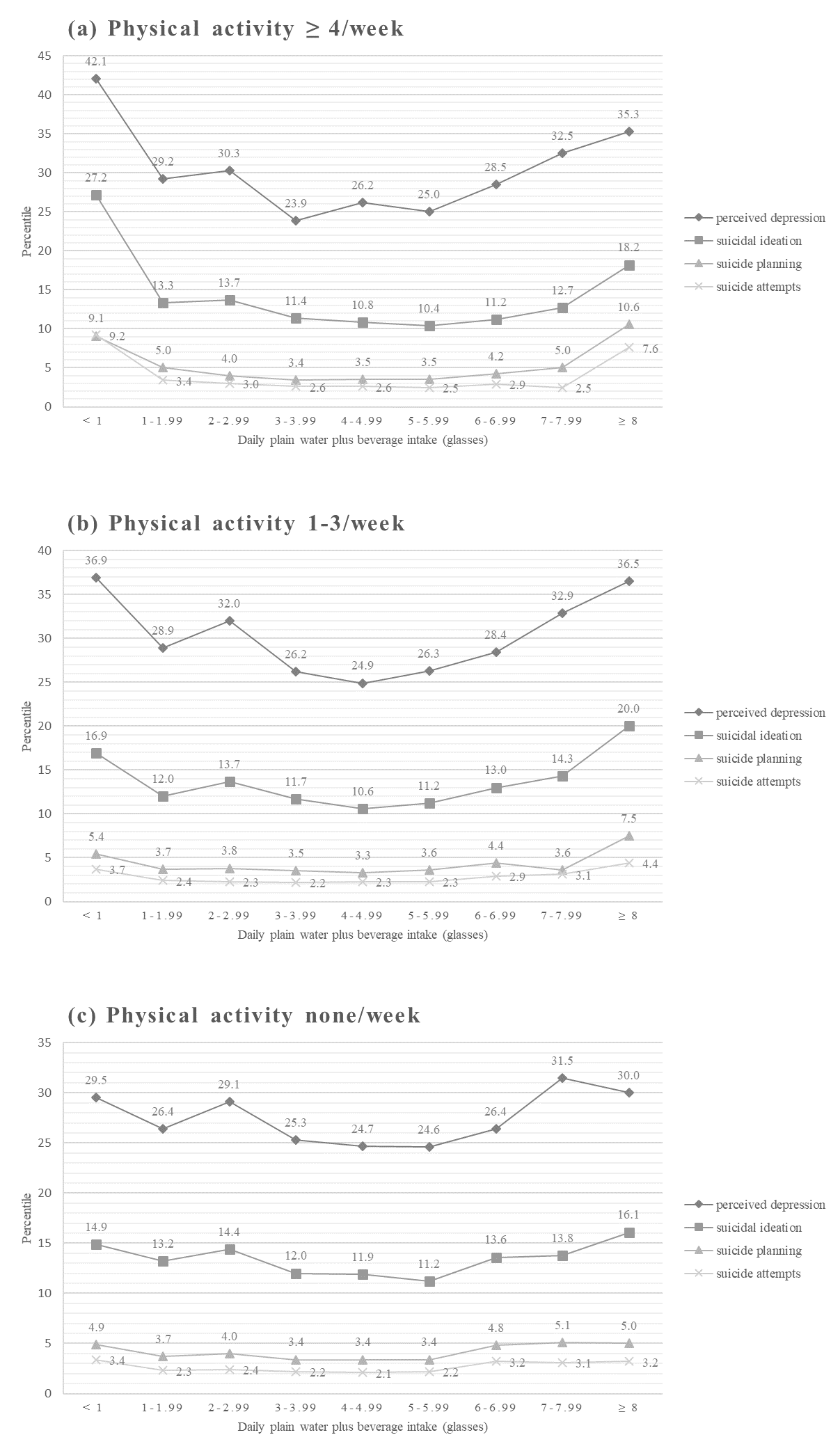

Supplement: Supplementary Material 6. — The weighted prevalences of perceived depression, suicidal ideation, suicide planning, and suicide attempts according to the daily water and beverage intake, stratified by physical activity. [file epih-46-e2024019-Supplementary-6.docx]
