# Supplementary material for: Association of plain water intake with self-reported depression and suicidality among Korean adolescents
Source: Epidemiol Health. 2024 Jan 9;46:e2024019. doi: 10.4178/epih.e2024019 (PMC11099597; doi:10.4178/epih.e2024019)
Supplement: Supplementary Material 7. — Association of beverage1 intake with perceived depression and suicidality. [file epih-46-e2024019-Supplementary-7.docx]

**Supplementary Material 7.** Association of beverage^1^ intake with perceived depression and suicidality.^2^

| Variable | Total  N = 112,250 | < 1 glass/day  n = 73,750 | 1-1.99 glasses/day  n = 30,758 | 2-2.99 glasses/day  n = 3,650 | 3-3.99 glasses/day  n = 2,162 | 4-4.99 glasses/day  n = 746 | ≥ 5 glasses/day  n = 1,184 |
| --- | --- | --- | --- | --- | --- | --- | --- |
| Perceived depression | 26.7 (0.2) | 25.0 (0.2) | 28.7 (0.3) | 32.8 (0.9) | 35.3 (1.1) | 34.2 (1.9) | 39.8 (1.5) |
| Suicidal ideation | 12.0 (0.1) | 11.1 (0.2) | 12.8 (0.2) | 14.9 (0.6) | 18.3 (0.9) | 14.7 (1.4) | 23.3 (1.4) |
| Suicide planning | 3.8 (0.1) | 3.4 (0.1) | 4.0 (0.1) | 4.7 (0.4) | 7.1 (0.6) | 5.8 (1.0) | 11.7 (1.0) |
| Suicide attempts | 2.5 (0.1) | 2.2 (0.1) | 2.6 (0.1) | 3.3 (0.3) | 4.8 (0.5) | 3.4 (0.7) | 8.9 (0.9) |

^1^Carbonated beverage plus sweetened beverage. ^2^Data are presented as weighted percentage (standard error).
