# Supplementary material for: Association of plain water intake with self-reported depression and suicidality among Korean adolescents
Source: Epidemiol Health. 2024 Jan 9;46:e2024019. doi: 10.4178/epih.e2024019 (PMC11099597; doi:10.4178/epih.e2024019)
Supplement: Supplementary Material 8. — Prevalences of perceived depression and suicidality according to the covariates. [file epih-46-e2024019-Supplementary-8.docx]

**Supplementary Material 8.** Prevalences of perceived depression and suicidality according to the covariates.^1^

|  | Perceived depression | *P* value | Suicidal ideation (%) | *P* value | Suicide planning (%) | *P* value | Suicide attempts (%) | *P value* |
| --- | --- | --- | --- | --- | --- | --- | --- | --- |
| Total | 26.7 (0.2) |  | 12.0 (0.1) |  | 3.8 (0.1) |  | 2.5 (0.1) |  |
| Sex |  |  |  |  |  |  |  |  |
| Male | 21.2 (0.3) |  | 8.7 (0.1) |  | 2.9 (0.1) |  | 1.6 (0.1) |  |
| Female | 32.7 (0.9) | < 0.001 | 15.5 (0.2) | < 0.001 | 4.7 (0.1) | < 0.001 | 3.4 (0.1) | < 0.001 |
| Type of school |  |  |  |  |  |  |  |  |
| Middle | 24.9 (0.3) |  | 12.2 (0.2) |  | 4.1 (0.1) |  | 2.8 (0.1) |  |
| High | 28.4 (0.3) | < 0.001 | 11.9 (0.2) | 0.264 | 3.4 (0.1) | < 0.001 | 2.2 (0.1) | < 0.001 |
| Economic status |  |  |  |  |  |  |  |  |
| High | 24.8 (0.3) |  | 10.4 (0.2) |  | 3.4 (0.1) |  | 2.2 (0.1) |  |
| Middle | 25,6 (0.3) |  | 11.1 (0.2) |  | 3.2 (0.1) |  | 2.0 (0.1) |  |
| Low | 36.8 (0.5) | < 0.001 | 20.3 (0.4) | < 0.001 | 7.1 (0.2) | < 0.001 | 5.1 (0.2) | < 0.001 |
| Academic achievement |  |  |  |  |  |  |  |  |
| High | 23.3 (0.3) |  | 10.6 (0.2) |  | 3.3 (0.1) |  | 2.0 (0.1) |  |
| Middle | 25.2 (0.3) |  | 10.7 (0.2) |  | 3.2 (0.1) |  | 2.1 (0.1) |  |
| Low | 31.9 (0.3) | < 0.001 | 14.9 (0.2) | < 0.001 | 4.9 (0.1) | < 0.001 | 3.5 (0.1) | < 0.001 |
| Physical activity |  |  |  |  |  |  |  |  |
| ≥ 4 days/week | 26.7 (0.3) |  | 11.4 (0.3) |  | 4.0 (0.1) |  | 2.9 (0.1) |  |
| 1-3 days/week | 27.3 (0.3) |  | 11.9 (0.2) |  | 3.7 (0.1) |  | 2.4 (0.1) |  |
| None | 26.0 (0.3) | < 0.001 | 12.5 (0.2) | < 0.001 | 3.7 (0.1) | 0.142 | 2.4 (0.1) | 0.001 |
| Smoking |  |  |  |  |  |  |  |  |
| None | 25.7 (0.2) |  | 11.4 (0.1) |  | 3.4 (0.1) |  | 2.1 (0.1) |  |
| Current | 43.4 (0.8) | < 0.001 | 22.8 (0.6) | < 0.001 | 9.4 (0.4) | < 0.001 | 8.6 (0.4) | < 0.001 |
| Alcohol consumption |  |  |  |  |  |  |  |  |
| None | 24.6 (0.2) |  | 10.8 (0.1) |  | 3.2 (0.1) |  | 1.9 (0.1) |  |
| Current | 40.9 (0.5) | < 0.001 | 20.5 (0.4) | < 0.001 | 7.6 (0.3) | < 0.001 | 6.3 (0.2) | < 0.001 |
| Carbonated beverage intake |  |  |  |  |  |  |  |  |
| None | 26.0 (0.3) |  | 12.2 (0.3) |  | 4.1 (0.1) |  | 2.6 (0.1) |  |
| ≤ 2/week | 25.4 (0.3) |  | 11.1 (0.2) |  | 3.3 (0.1) |  | 2.1 (0.1) |  |
| 3-6/week | 28.0 (0.3) |  | 12.5 (0.2) |  | 3.7 (0.1) |  | 2.6 (0.1) |  |
| daily | 31.7 (0.6) | < 0.001 | 15.2 (0.5) | < 0.001 | 6.1 (0.3) | < 0.001 | 4.3 (0.3) | < 0.001 |
| Sweetened beverage intake |  |  |  |  |  |  |  |  |
| None | 24.0 (0.4) |  | 11.4 (0.3) |  | 3.9 (0.2) |  | 2.6 (0.1) |  |
| ≤ 2/week | 24.6 (0.3) |  | 10.8 (0.2) |  | 3.2 (0.1) |  | 2.2 (0.1) |  |
| 3-6/week | 27.7 (0.3) |  | 12.3 (0.2) |  | 3.7 (0.1) |  | 2.4 (0.1) |  |
| daily | 34.3 (0.5) | < 0.001 | 13.7 (0.3) | < 0.001 | 5.8 (0.2) | < 0.001 | 4.0 (0.2) | < 0.001 |
| Plain water consumption |  |  |  |  |  |  |  |  |
| ≥ 3 glasses/d | 25.7 (0.2) |  | 11.4 (0.1) |  | 3.6 (0.1) |  | 2.4 (0.1) |  |
| 1-2 glasses/d | 29.0 (0.4) |  | 13.2 (0.3) |  | 3.9 (0.2) |  | 2.5 (0.1) |  |
| < 1 glass/d | 36.4 (0.8) | < 0.001 | 19.0 (0.6) | < 0.001 | 6.7 (0.4) | < 0.001 | 4.8 (0.4) | < 0.001 |

^1^Data are presented as weighted percentage (standard error).
